# Mindfulness Improves Awareness and Cortisol Levels During COVID-19 Lockdown: A Randomised Controlled Trial in Healthcare Workers

**DOI:** 10.3390/healthcare13192455

**Published:** 2025-09-27

**Authors:** Anna Panzeri, Ornella Bettinardi, Fabio Giommi, Maddalena Grassi, Massimo Rossetti, Pasqua Barile, Barbara Del Bello, Concetta Gardi

**Affiliations:** 1 Department of General Psychology, University of Padua, 35100 Padua, Italy; anna.panzeri@unipd.it; 2 Department of Mental Health and Pathological Addiction, Piacenza Local Health Authority, 29121 Piacenza, Italy; o.bettinardi@ausl.pc.it (O.B.);; 3 NOUS-School of Specialization (PsyD) in Cognitive and Mindfulness-Oriented Psychotherapy, 20137 Milano, Italy; fabiomario.giommi@gmail.com; 4 Department of Molecular and Developmental Medicine, University of Siena, 53100 Siena, Italy; barbara.delbello@unisi.it

**Keywords:** attention and awareness, clinical psychology, mindfulness, randomized controlled trial, stress biomarkers

## Abstract

**Background**: During the coronavirus disease 2019 (COVID-19) pandemic, healthcare professionals (HCPs) faced extremely high levels of work-related stress, which negatively affected their mental health, job satisfaction, and patient care. Mindfulness-Based Stress Reduction (MBSR) programs can help mitigate these challenges. **Method**: A randomised controlled trial design was used to evaluate the effects of an 8-week MBSR intervention on stress-related outcomes among HCPs compared with a control group. The comprehensive assessment included cortisol levels, perceived stress, attention and awareness, emotional fatigue, and job strain. **Results**: The MBSR intervention significantly reduced cortisol levels in the short term and improved attention and awareness, with sustained benefits during follow-up. Medium to medium–large effect sizes were observed for job strain and emotional fatigue, although statistical significance was not achieved. Similarly, perceived stress showed negligible changes over time. The variability in stress responses highlights the importance of personalised approaches to mindfulness interventions. **Discussion**: In the context of the COVID-19 pandemic, MBSR is promising for improving attention and awareness and reducing physiological stress markers in HCPs, although its effects on other occupational outcomes remain uncertain. Future research should focus on longer interventions to maximise the benefits of mindfulness practices in healthcare settings.

## 1. Introduction

The COVID-19 pandemic has dramatically increased the workload of healthcare professionals (HCPs), exacerbating the already high levels of stress, with significant consequences for both physical and psychological well-being [1,2,3]. In the first period of the pandemic, HCPs were forced to reconsider their priorities and face difficult and unexpected situations. Since the beginning, they have been committed to fighting on the front line against the epidemic and protecting public health. In this context of emergencies, healthcare operators have experienced a significant increase in workload, a high risk of infection, and strong work pressure. Continuous exposure to emergencies due to COVID-19 has created a prolonged situation of work-related stress that has led to increased burnout syndrome in HCPs [4,5,6]. A study conducted during the pandemic on 2707 health operators from 60 countries reported that 51% of them experienced burnout [7]. Burnout in HCPs during the pandemic was associated with four main professional risk factors: limited organisational support, high levels of workload, work stress, and having to work in situations of high emergency [7].

All these factors, due to operator burnout, may also have affected the patients, negatively impacting the quality of care, reducing adherence to the treatment provided, and worsening outcomes related to the disease. A study conducted on 933 healthcare workers from various regions of Italy [8] revealed that up to 25% of HCPs experienced clinically significant levels of work-related burnout during the pandemic, with over 50% exhibiting symptoms of depression, over 60% showing symptoms of anxiety, and over 50% experiencing symptoms of post-traumatic stress. Overall, more than half of the Italian HCPs who participated in the study reported experiencing high levels of psychological distress. Additionally, the authors reported that HCPs who experienced alarming levels of burnout were those who had worked extensively on the front lines and had experienced the loss of multiple patients [8]. These HCPs had fewer personal resources available and suffered more from depression, anxiety, and post-traumatic symptoms compared to those with lower burnout levels [8,9,10]. These findings corroborate data from previous research, which highlighted that close patient contact can be a significant factor in burnout [11].

Among HCPs, mental health care professionals (M-HCPs) were uniquely impacted. Unlike their peers, who focused on physical symptoms and emergency interventions, M-HCPs were tasked with addressing the psychological fallout of the pandemic [12,13,14]. Their role involved supporting individuals struggling with heightened anxiety, depression, grief, and trauma, conditions exacerbated by isolation, uncertainty, and social disruption [15]. In fulfilling these responsibilities, M-HCPs encountered distinct psychological burdens, including prolonged emotional strain, elevated burnout risk, and vicarious trauma [16,17,18]. They also extended support to fellow healthcare workers, particularly those on the front lines, who themselves faced intense psychological challenges [16,19]. These compounded demands suggest that M-HCPs may experience stress responses that differ in nature and intensity from those of other healthcare groups, underscoring the need for targeted research and tailored interventions.

Despite the many adverse psychosocial effects of the pandemic, the presence of protective factors has shown promise in buffering stress and promoting resilience [20]. Providing adequate support and developing effective tools to address emerging symptoms has become essential. Among these, Mindfulness-Based Interventions (MBIs), particularly MBSR, have gained attention for their potential to enhance mental health and well-being among healthcare workers facing extreme stress [21,22,23].

MBSR is a program that teaches mindfulness skills [24] and helps individuals cope with stress. This ability has proven beneficial in reducing stress and encouraging individuals to engage in mindful behaviours that are more effective in managing stress reactions [25]. Through mindfulness, individuals cultivate greater tolerance for painful feelings, and the level of automatic reactivity to negative events is diminished. Mindfulness is a method based on working with one’s body, breathing, and observing emotions and thoughts. This practice increases awareness of one’s resources (e.g., self-esteem), enhances mindfulness, and improves concentration during daily activities, resulting in more effective and aware stress responses [24,26]. This contributes to improved well-being, psychophysical balance, and an overall higher quality of life. Wexler and Schellinger [23] conducted an integrative review of 833 articles on the effects of MBIs on nurses during the COVID-19 pandemic. The authors reported that 60% of the studies demonstrated a significant impact of the mindfulness program on reducing stress, decreasing burnout, and increasing awareness. MBIs were also effective in alleviating anxiety, improving sleep quality, and enhancing awareness in frontline nurses at the initial epicentre of the pandemic [27]. During the pandemic, MBIs were also offered online, making participation in these courses more accessible [28,29]. For example, a brief online mindfulness intervention effectively reduced fatigue and burnout among nurses of geriatric patients, with long-lasting effects [30].

Emerging evidence suggests that MBIs may also influence biological stress markers, particularly cortisol [31,32]. Stress activates the hypothalamic–pituitary–adrenal–(HPA) axis, leading to elevated levels of glucocorticoids, such as cortisol, a key biomarker of physiological stress [33]. Chronic HPA activation is associated with persistent inflammation and a range of physical and mental health disorders [34,35,36].

Individual differences in stress perception and coping strategies modulate this response [37,38]. Mindfulness practices, by fostering nonreactive awareness, may reduce autonomic arousal and prevent nervous system dysregulation. Enhanced emotional regulation through mindfulness is linked to lower stress and anxiety levels, and neuroimaging studies have shown that mindfulness can alter brain regions involved in emotion regulation and HPA axis control [39,40].

This study responds to the urgent need to address the psychological and physiological toll of the pandemic on M-HCPs, a subgroup of healthcare professionals exposed to uniquely intense stressors. While MBIs have shown promise across general healthcare settings, research specifically targeting M-HCPs and examining both psychological and biological outcomes (e.g., cortisol) remains limited. Against this backdrop, the present study aims to evaluate the potential protective effects of an eight-week MBSR program on psychological well-being and cortisol levels in M-HCPs exposed to prolonged stress before and after the onset of the COVID-19 pandemic.

## 2. Materials and Methods

An intervention design with randomisation was employed, resulting in a open-label randomised controlled trial (RCT). The sampling list was composed of individuals who expressed their willingness to participate in the MBSR intervention. All names were recorded in a list according to the order of their registration. A simple randomisation sequence was generated via the online program Random Number Generator [41] by a nurse who was not involved in participant recruitment, intervention delivery, or outcome assessment for the present study. To ensure allocation concealment, the randomisation list was accessible only to the Principal Investigator (PI) of the study. Based on the principle of randomisation, the participants were assigned to two groups: the experimental group participated in an MBSR intervention (see dedicated section), whereas the control group received no intervention. All participants (both experimental and control groups) underwent psychological and physiological assessments at three time points: before the intervention (T0), after the 8-week intervention (T1, postintervention), and at the six-month follow-up (T2). The primary outcome was assessed at T1 (postintervention).

**Figure 1:**
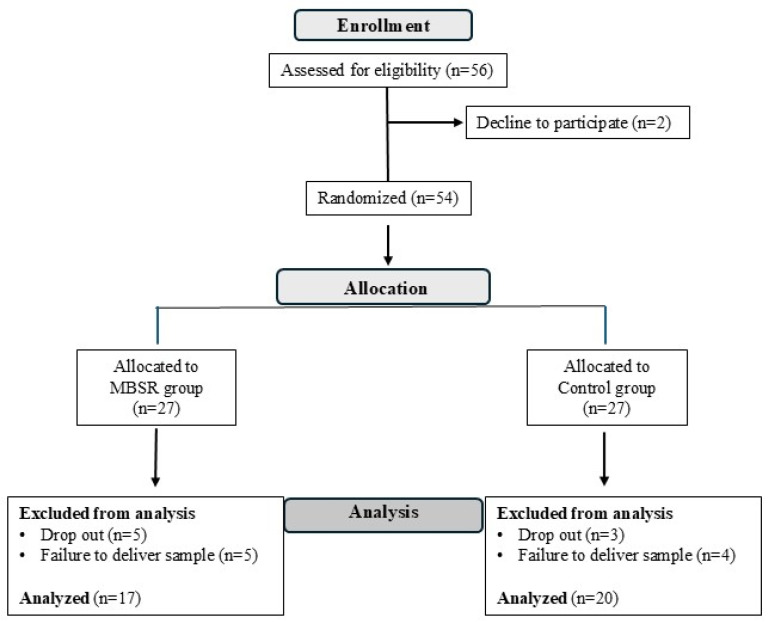
CONSORT flow diagram for participants in our study.

Figure 1 displays the CONSORT flow diagram. This includes enrolment, allocation, and analysis of participants. After screening for eligibility, 54 subjects were randomly allocated to groups. After the 8-week intervention, 10 participants from the MBSR group and 7 from the control group were excluded from the analysis due to either failure to provide a saliva sample or dropout.

### 2.1. Participants and Procedure

This study was conducted during the second phase of the lockdown (between October and December 2020) in Emilia Romagna, one of the most affected regions of northern Italy, where COVID-19 resulted in more than 700 deaths during the first phase of the lockdown between March and May 2020. For this study, 56 healthcare professionals (M-HCPs) working in the Local Health Care District of Piacenza (Northern Italy) were recruited. The inclusion criteria were as follows: operators from the department mentioned above aged between 18 and 65 years. The exclusion criteria included the use of psychotropic drugs in the last two months, previous participation in mindfulness training, unwillingness to sign the informed consent form, medical conditions preventing participation in the groups (including endocrine disorders, current psychiatric treatment, and the use of corticosteroid medication, which could confound cortisol measurements), and current pregnancy status.

The study started after approval was obtained from the Ethical Committee of Area Vasta Nord (region Emilia Romagna, Italy) (n° 2020/GCLIN/0014), and written informed consent was obtained from all participants. This study was registered on ClinicalTrials.gov (acceseed on 25 July 2025) (identifier: NCT07129057).

### 2.2. Description of the MBSR Intervention

The MBSR program is an introductory course to mindfulness practices that involves the systematic and continuous cultivation of specific mental states, promoting a different relationship with stress and discomfort [24]. The program lasts eight consecutive weeks (two months), with group sessions lasting two and a half hours each and an intensive, full-day, 8-h practice session. At each session, the participants received audio tracks for daily home practice.

### 2.3. Physiological Evaluation

To assess cortisol levels, at the experimental times (T0, T1, and T2), saliva samples were collected using Salivettes (Sarstedt) 30 min after waking up. The participants were instructed not to drink, eat, chew gum, or brush their teeth within the 30 min preceding sample collection. To collect the saliva, participants were advised to rinse their mouth with water for 30 s, wait 10 min, and then remove the swab from the Salivette and chew it for approximately 2 min to stimulate salivation. The swab was reinserted into the Salivette, frozen at −20 °C, and stored at the AUSL Piacenza Analysis Laboratory before being shipped to the University of Siena for further analysis. Upon arrival at the Siena laboratory, the samples will be thawed and centrifuged at 2000× *g* for 5 min at 4 °C. Salivary cortisol levels were assayed using an Enzyme Immunoassay kit (Arbor Assays Inc., Ann Arbor, MI, USA).

### 2.4. Psychological Evaluation

Beyond a socio-demographic schedule registering basic information such as age, sex, and occupational role, the psychological evaluation was carried out through the following self-report assessment tools validated in the Italian language:

The Perceived Stress Scale (PSS) [42] measures the degree to which situations in a person’s life are appraised as stressful. Each item was rated on a scale ranging from 0 (never) to 4 (very often). The total score ranges from 0 to 40, with higher scores indicating a greater degree of subjective stress. A total score of 0–13 was considered low perceived stress, 14–26 was considered moderate perceived stress, and 27–40 was considered high perceived stress.

The Italian Cardiologists’ Undetected Distress Study (IANUS) [43] consists of 15 items to measuring job strain, positive meaning, emotional fatigue, and relational difficulties in the workplace. Items are scored on a Likert-type response scale (never = 1; sometimes = 2; often = 3; almost always = 4), and the scores of each subscale range from 0–100.

The Mindfulness Awareness Attention Scale (MAAS) [44] is a 15-item self-report instrument investigating awareness and attention to what is happening in the present. This questionnaire measures dispositional mindfulness, specifically the frequency of open or receptive attention to and awareness of present-moment experiences. Participants rated each item (e.g., “I find it difficult to stay focused on what’s happening in the present”) on a 6-point Likert scale ranging from 1 (almost always) to 6 (almost never), with higher scores indicating greater mindfulness. The MAAS has demonstrated high internal consistency and good psychometric properties in both clinical and nonclinical populations.

### 2.5. Qualitative Observations

Due to time and organisational constraints related to implementing the MBSR protocol and gathering biological data during the pandemic, the qualitative part of the study was limited to direct observations and focus groups. Observational data were collected during weekly MBSR sessions by trained facilitators who documented participant engagement, verbal and non-verbal responses, and group dynamics. These observations provided contextual insights into how participants interacted with the mindfulness practices and with each other.

### 2.6. Statistical Data Analysis

Descriptive statistics were used to describe the characteristics of the sample in terms of sociodemographic characteristics and clinical variables over time. Student’s *t*-test and chi-square test were used to test the homogeneity of the control and experimental groups, respectively, for continuous and categorical dependent variables. Cohen’s d was used as an effect size for the *t*-test, as its significance may be influenced by sample size. Repeated measures analysis of variance (ANOVA) was used to evaluate the levels of clinical variables over time (T0, T1, T2) across the two groups (control group and experimental MBSR group). The type III decomposition of variance was used. Partial eta squared (η^2^ₚ) was used as an effect size in ANOVA to indicate the proportion of variance explained by an independent variable relative to the total variance, excluding other factors. The general interpretation of η^2^ₚ follows Cohen’s [42] guidelines for effect sizes: 0.01 (1%) small effect; 0.06 (6%) medium effect; and 0.14 (14%) large effect. Post hoc comparisons were used when statistically significant effects or medium-to-large effect sizes emerged. To correct for multiple testing in post hoc comparisons, the *p*-values were corrected with the Holm method. Jamovi software (version 2.6.26), which is based on R, was used for all the statistical analyses. The alpha level was set at 0.05.

## 3. Results

### 3.1. Sample Characteristics

Thirty-seven participants, 31 females and 6 males, with a mean age of 42.24 years (SD 11.07) (range 25–63 years), completed all the measurements (Figure 1). Most respondents were nurses and healthcare assistants (82%), and the others were doctors and psychologists (18%). The work setting was equally balanced between hospital departments (n = 20, 53%) and territorial services (n = 18, 47%). Table 1 shows the participants’ sociodemographic characteristics.

### 3.2. Baseline Homogeneity Between the Two Groups

The participants were randomly allocated to the experimental MBSR group or the control group, which did not receive any intervention. All data from participants who completed all the measurement points were analysed (Figure 1). At T0, the control and experimental MBSR groups were homogeneous in terms of age (t = |0.29|, df = 35, *p* = 0.773, Cohen’s d = −0.10, negligible effect size), sex (X^2^ = 0.05, df = 1, *p* = 0.828), work position (X^2^ = 0.07, df = 1, *p* = 0.791) (Table 1), and physiological and psychological characteristics (Table 2).

### 3.3. Repeated-Measures ANOVA

Below are reported the results of the repeated measures ANOVAs on the outcomes.

#### 3.3.1. Cortisol Outcome

**Figure 2:**
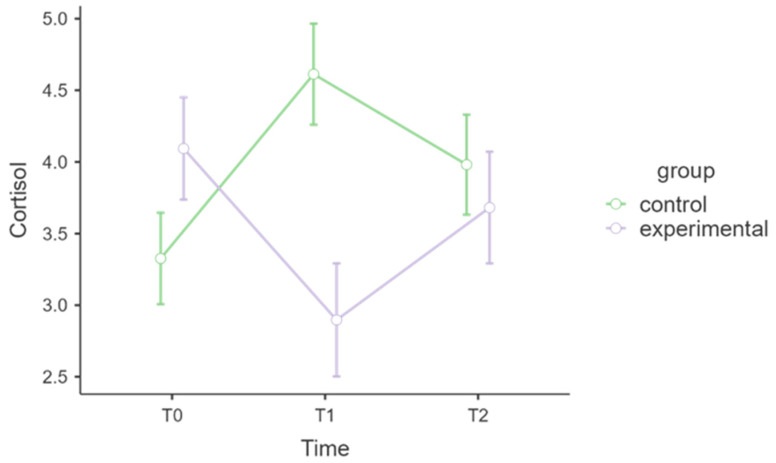
Estimated marginal means for cortisol.

Figure 2 displays different cortisol trajectories over time across groups.

Table 3 indicates that the interaction between time and group was statistically significant, with a very large effect size (F = 10.08, *p* < 0.001, η^2^ₚ = 0.23). In the experimental group, cortisol levels decreased from T0 to T1; meanwhile, the cortisol scores of the control group increased from T0 to T1. By T2, both groups had similar scores. The post hoc comparisons for cortisol indicated that at T1, the experimental group, after the MBSR intervention, presented significantly lower cortisol levels than did the control group, which did not undergo the MBSR intervention (t = 3.24, *p* = 0.003, p-holm = 0.037).

#### 3.3.2. MAAS, Attention, and Awareness

**Figure 3:**
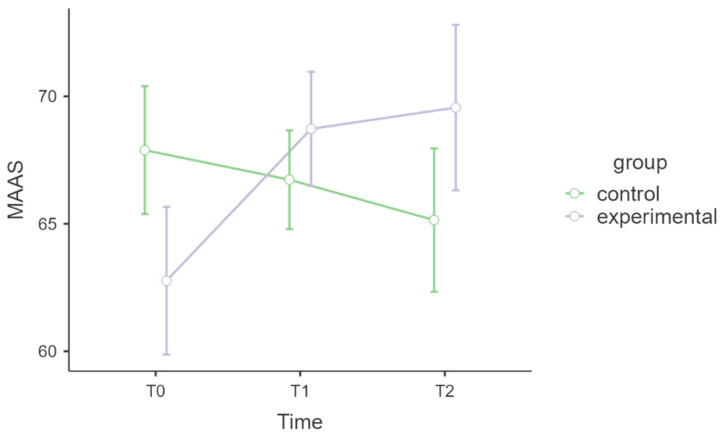
MAAS, attention and awareness.

Table 4 shows the model regarding attention and awareness. The interaction of time ✻ group was statistically significant (F = 4.51, *p* = 0.015, η^2^_p_ = 0.12), meaning that the two groups had different levels of attention and awareness over time. In the post hoc comparisons, the corrected *p*-values revealed no statistically significant differences. The noncorrected *p*-values were significantly different in the experimental group from T0 to T1 (t = 2.60, df = 33, *p* = 0.014) and from T0 to T2 (t = 2.35, df = 33, *p* = 025), suggesting that the MAAS scores increased in the MBSR group but not in the control group. Figure 3 shows the graph of MAAS levels.

#### 3.3.3. Perceived Stress Levels

**Figure 4:**
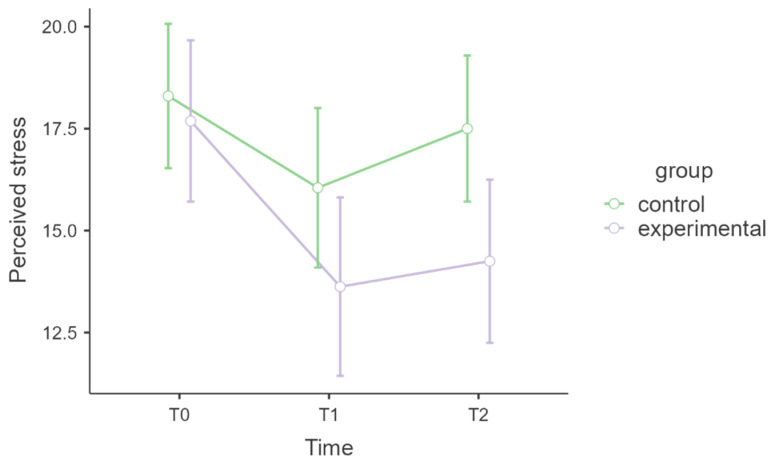
Perceived stress over time and across groups.

Table 5 shows the model for perceived stress. No statistically significant effect of the interaction time * group emerged (*p* = 0.471), and the associated effect size was small (η^2^ₚ = 0.02). Figure 4 shows a graph of the levels of perceived stress.

#### 3.3.4. IANUS, Job Strain

**Figure 5:**
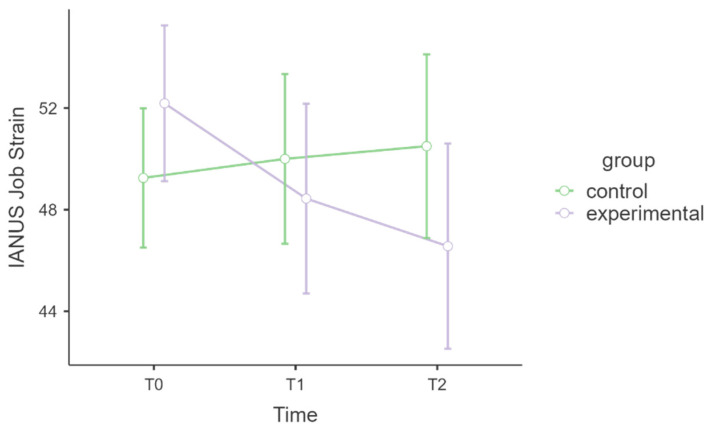
IANUS Job Strain.

Below, the relevant results of the IANUS (job strain and emotional fatigue) are reported, as the scales of positive meaning and relational difficulties did not provide statistically significant results, probably because they were not specifically targeted by the MBSR intervention. Table 6 shows the model for the IANUS job strain subscale. The interaction of time * group was not statistically significant (F = 1.74, df = 2, *p* = 0.183), but the associated effect size was medium (η^2^ₚ = 0.05). This suggests that the two groups appear to follow almost different trajectories over time, but do not reach statistical significance. The post hoc comparisons revealed no statistically significant effect. Figure 5 shows a graph with the levels of IANUS job strain.

#### 3.3.5. IANUS Emotional Fatigue

**Figure 6:**
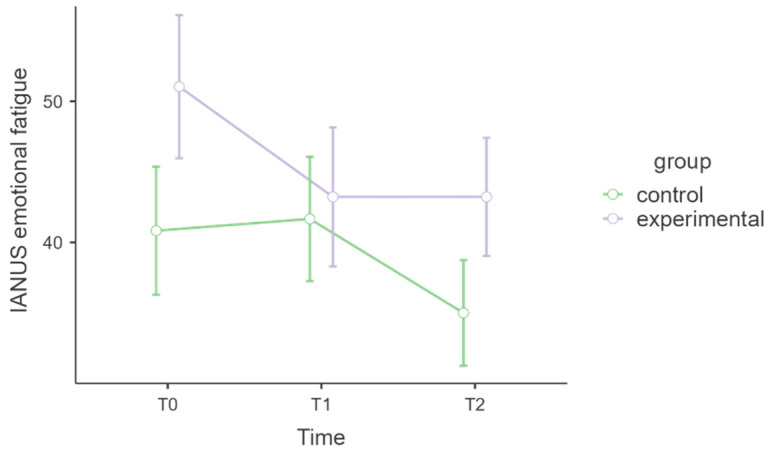
IANUS emotional fatigue.

Table 7 shows the emotional fatigue model; the interaction time * group was not statistically significant (*p* = 0.059), but the associated effect size was medium (η^2^ₚ = 0.08). Post hoc comparisons revealed that the experimental MBSR group significantly improved from T0 to T1 (t = 3.13, df = 34, *p* = 0.004). Figure 6 shows a graph of the levels of emotional fatigue.

### 3.4. Qualitative Observations Results

Throughout the sessions, instructors consistently observed a high level of engagement, attentiveness, and active participation among the participants. As the program advanced, notable improvements were evident in participants’ concentration, self-awareness, and their ability to manage stress and regulate emotions. Insights gathered from focus groups further underscored the positive impact of the intervention. Participants reported meaningful enhancements in stress management, personal insight, and overall quality of life. The skills developed during the program were perceived as beneficial not only in professional contexts but also in everyday life, contributing to a greater sense of well-being and personal growth. Mindfulness practices were widely regarded as effective tools for cultivating deeper self-connection and interpersonal awareness, helping to alleviate feelings of isolation.

## 4. Discussions

This study highlights the urgent need to investigate the psychological and physiological effects of the pandemic on M-HCPs, a subgroup of healthcare professionals exposed to uniquely intense stressors. While MBIs have shown potential across various healthcare settings, research specifically focused on M-HCPs evaluating both psychological and biological outcomes (e.g., cortisol) remains limited. This study investigated the potential protective effects of an eight-week MBSR programme on cortisol levels and the psychological well-being among M-HCPs within an Italian healthcare district exposed to prolonged stressful conditions before, during, and after the COVID-19 pandemic.

A significant reduction in cortisol levels was observed in the experimental group following the intervention, with a notable effect size compared with that of the control group. Cortisol, a biomarker of physiological stress, exhibited different patterns between groups. While the MBSR group experienced a significant decrease in cortisol from T0 (4.09 ± 1.60) to T1 (2.90 ± 1.14), the control group showed an increase (T0: 3.33 ± 1.27; T1: 4.61 ± 1.85). This pattern suggests that practising MBSR may have helped reduce physiological stress during the COVID-19 pandemic, as reflected by salivary cortisol levels. These findings align with previous research indicating that mindfulness practices can downregulate HPA axis activity and lower stress-related hormonal responses [32,45]. Cortisol can directly impact brain regions involved in stress processing and emotional regulation [46]. MBSR has been shown to reduce amygdala reactivity and suppress cortisol secretion, thereby enhancing emotional resilience [39,47,48].

A meta-analysis confirmed the effectiveness of meditation in reducing cortisol levels, particularly in high-stress populations, supporting the recommendation for extended interventions in stressful contexts [49].

However, the effect was not maintained at follow-up, implying that ongoing mindfulness practice might be necessary to sustain physiological stress reductions, an idea supported by longitudinal mindfulness studies [50,51]. The reductions in cortisol after the intervention and their partial persistence at follow-up highlight the potential of mindfulness-based approaches to help regulate stress during periods of high psychosocial challenge, such as the COVID-19 pandemic.

Self-reported stress, measured by the PSS, decreased more in the MBSR group (T0: 17.76 ± 6.95; T1: 13.62 ± 8.28) than in the control group (T0: 18.30 ± 8.46; T1: 16.05 ± 9.11). Despite small increases at follow-up in both groups, the MBSR group maintained lower overall stress levels. However, the MBSR intervention did not produce a statistically significant difference in perceived stress over time compared with the control group, with a small effect size. Importantly, the stress levels of the HCPs in our study varied widely. While some research reports short-term reductions in perceived stress [52], others indicate high variability and limited long-term effects, especially when baseline stress levels are already high [53,54]

Notably, improvements in attention and awareness persisted beyond the intervention, demonstrating MBSR’s ability to promote lasting benefits in terms of self-monitoring, being fully present, aware and focused. Additionally, these findings align with studies documenting sustained increases in self-awareness and attentional control following regular mindfulness practices. A thematic analysis revealed that participants continued to report enhanced self-awareness, emotional regulation, and cognitive clarity up to three years post-MBSR, particularly when they maintained regular practice [51]. These findings suggest that the attentional and self-awareness benefits of MBSR may be more pronounced and enduring than its physiological effects. The lasting benefits in mindfulness-related attention and awareness are well supported by an Italian healthcare study where MAAS scores increased significantly from 64.64 to 72.25 (*p* = 0.027), indicating durable cognitive effects even without sustained cortisol changes [52]. The medium effect size observed in the present study underscores the clinical significance of these attentional and awareness improvements in the context of work-related stress.

Some IANUS subscales showed improvements in occupational well-being among MBSR participants. Although the interaction effects for job strain and emotional fatigue did not reach statistical significance, medium to large effect sizes were observed. The participants in the MBSR group exhibited trends towards decreased job strain and emotional fatigue postintervention and at follow-up. These patterns align with prior studies suggesting MBSR’s potential to mitigate emotional exhaustion in high-stress work environments, even when statistical significance is not achieved, possibly due to limited sample sizes or high baseline variability [52,54,55]. Conversely, the intervention did not impact positive meaning or relational difficulties. High variability among participants and small effect sizes may reflect unmeasured external stressors or organisational factors that are less amenable to change through individual-level interventions such as MBSR.

Overall, these findings demonstrate that MBSR intervention positively impacted cortisol levels and improved attention and awareness. A promising trend towards reductions in stress, job strain, and emotional fatigue emerged, although further research is necessary to establish the extent and stability of these effects.

### 4.1. Limitations and Future Research

This study has several limitations that should be acknowledged, as they may affect the interpretation and generalisability of the findings. A limitation of this study is the relatively small final sample size (n = 37), which was constrained by the limited eligible population available at our centre during the COVID-19 pandemic (Supplementary Materials). While all consecutive participants during the recruitment period were included, the limited sample size reduces the statistical power to detect significant effects, especially for outcomes with medium or small effect sizes, increasing the risk of missing meaningful effects (e.g., type II error) and restricting the generalizability of the findings. High attrition rates further exacerbated this issue (61% of the original sample completed the study). The dropout rate in this study, although relatively high, aligns with attrition rates commonly reported in clinical and behavioural research (10–25%). Factors contributing to dropout may include participant motivation and logistical hurdles (e.g., scheduling and travel demands). These challenges highlight the need for careful consideration of retention strategies in future trials (e.g., flexible scheduling, remote participation, and enhanced participant engagement) to improve adherence and reduce attrition. Thus, these findings should be interpreted with caution due to these constraints. Together, these limitations underscore the necessity for larger, more rigorously designed trials to confirm and extend the present findings. Future research should aim to recruit larger samples and account for potential dropouts by oversampling. Moreover, the absence of an active control group (e.g., relaxation training) limits the ability to attribute observed effects specifically to MBSR intervention; future research should incorporate active comparators to strengthen causal conclusions. Psychological outcomes were measured by self-reported scales, which are widely used but are prone to biases (e.g., desirability, recall inaccuracies). Using hetero-reported clinical assessments would provide a more comprehensive understanding of the effects of MBSR. The absence of an active control group (e.g., progressive relaxation, cognitive-behavioural therapy) makes it difficult to distinguish the specific effects of MBSR from nonspecific factors (e.g., group support, facilitator attention). Future studies should include active control conditions to better assess the unique contributions of MBSR. At preintervention, the experimental and control groups were not always balanced in terms of dependent variables (e.g., stress levels, emotional fatigue), which could confound the results, as differences observed after the intervention may partly reflect preexisting disparities rather than the effects of the intervention. Future RCTs with stratified randomisation based on baseline characteristics could address this issue. One notable limitation of the present study is the predominant representation of female participants, reflecting a broader trend in MBI research, where women are more likely to engage in psychological and wellness programs. This imbalance may introduce gender-related bias, as emotional regulation strategies and responsiveness to mindfulness practices can differ by gender [56]. As a result, the predominance of female participants may constrain the generalizability of findings to male or gender-diverse healthcare professionals. Future studies should pursue more balanced gender recruitment to explore differential outcomes better and strengthen external validity. Potential covariates that might influence outcomes (e.g., workplace stressors, individual differences in coping strategies, social support, engagement with mindfulness outside formal sessions) were not considered. Future research should track these additional variables to adjust for their possible influence, also focusing on the protective factors such as self-esteem, coping, social support, and the forgiveness of the situation [37,57,58,59,60,61]. Moreover, additional statistical models, such as mediation analysis, in longitudinal study could further investigate the mechanisms through which MBSR works [27,62,63]. By addressing these methodological challenges, future research can provide stronger evidence for the efficacy of MBSR and refine its implementation across various settings.

### 4.2. Strengths of the Study

This study presents several notable strengths that enhance its contribution to the expanding literature on MBSR in healthcare settings. By focusing on M-HCPs, a subgroup particularly vulnerable to occupational stress and burnout during the COVID-19 pandemic [45,46], this research addresses a critical gap in the current evidence base. M-HCPs often face sustained emotional demands, making them an ideal population for evaluating the therapeutic potential of mindfulness interventions. While previous studies have demonstrated the efficacy of MBSR in reducing psychological distress among healthcare workers [32,48], this study adds originality by incorporating biological markers, specifically salivary cortisol, to assess physiological stress responses. The inclusion of cortisol as a biomarker not only strengthens the methodological rigour but also aligns with emerging research emphasising the importance of evaluating both psychological and physiological outcomes in mindfulness studies [47,49]. This dual approach enhances the relevance of the findings and supports the broader application of MBSR in high-stress clinical environments. The use of an RCT design, regarded as the gold standard for evaluating intervention efficacy, minimises biases, ensures comparability between groups, and increases the reliability of the findings. Randomisation enhances the internal validity and allows causal inferences about MBSR’s effects. The study’s comprehensive assessment of multiple outcomes, including cortisol levels, attention and awareness, perceived distress, emotional fatigue, job strain, positive meaning, and relational difficulties, offers a holistic understanding of MBSR’s impact and aligns with previous research showing its broad benefits for mental health, stress reduction, and overall well-being [49,51,54,64]. The longitudinal design, with assessments conducted at preintervention, postintervention, and follow-up, enables the capture of both immediate and sustained effects of the intervention during the COVID-19 pandemic, underscoring the time-varying nature of mindfulness-related benefits [51,65].

An additional strength is the presence of both qualitative and quantitative insights, as the study complements existing findings on the transformative effects of MBSR, particularly with respect to stress-related outcomes. Despite the absence of a formal thematic analysis due to resource constraints, the study benefited from preliminary coding of transcripts and field notes, which enabled the identification of recurring patterns and experiential themes. This approach enriched the interpretation of the quantitative data by offering complementary qualitative insights, thereby enhancing the overall understanding of the subjective impact of the MBSR program. The integration of observational and focus group data provided valuable context and depth, capturing nuances in participant experiences that might otherwise have been overlooked. This dual approach strengthens the evidence base. The intervention adhered to the standardised 8-week MBSR protocol, ensuring consistency with established practices and enabling comparability. Despite some nonsignificant results, medium to large effect sizes in trends suggest promising avenues for further investigation, demonstrating that mindfulness interventions may offer benefits beyond statistical significance. Overall, by evaluating multiple outcomes in a high-stress population through a robust longitudinal design, this study makes a valuable contribution to understanding the efficacy and applicability of MBSR in clinical and occupational contexts.

## 5. Conclusions

This study advances the understanding of the specific needs of M-HCPs by addressing both the psychological and physiological dimensions of stress within a subgroup exposed to uniquely intense occupational pressures, particularly during crises such as the COVID-19 pandemic. Whereas most prior research on M-HCPs has focused primarily on self-reported stress, the present study offers preliminary evidence of the short-term efficacy of an MBSR intervention in lowering cortisol levels, suggesting immediate physiological benefits for individuals operating in high-stress environments. These findings underscore the potential of MBSR to achieve rapid stress-reducing effects and support the need for larger, adequately powered trials to evaluate its effectiveness rigorously. Beyond physiological outcomes, the intervention yielded sustained improvements in attention and awareness, reinforcing its cognitive benefits, as documented in earlier research [66]. The study also highlights the importance of accounting for individual variability in stress responses to better tailor mindfulness-based interventions. Although emotional fatigue, job strain, and perceived stress demonstrated positive trends, these outcomes did not reach statistical significance, likely because of methodological constraints such as small sample sizes and high baseline variability. Future research should address these limitations by recruiting larger and more diverse samples, employing stratified randomisation, and incorporating active control conditions to isolate the specific effects of MBSR.

Overall, this study provides valuable evidence supporting MBSR as a promising intervention for healthcare professionals experiencing chronic stress. By demonstrating both physiological and cognitive benefits, this study lays the groundwork for more targeted, long-term investigations and reinforces the biological plausibility of mindfulness-based approaches as effective tools for stress management in high-pressure occupational settings.

## Figures and Tables

**Table 1:** Baseline characteristics of the sample stratified by group.

Variables	Control Group (n = 20)	MBSR Group (n = 17)	Statistics and *p* Value
Age (Mean ± SD)	41.75 ± 10.93	42.82 ± 11.54	t = |0.29|, df = 35, *p* = 0.773, Cohen’s d = −0.10
*Sex* Male Female	3 17	3 14	X^2^ = 0.05, df = 1, *p* = 0.828
*Work position* nurses, educators, and social health workers	16	14	X^2^ = 0.07, df = 1, *p* = 0.791
physicians, psychologists	4	3	

**Table 2:** Descriptive statistics of the psychological and physiological variables.

	Control Group (*n* = 20)	Experimental MBSR Group (*n* = 17)
Cortisol t0	3.33 (1.27)	4.09 (1.60)
Cortisol t1	4.61 (1.85)	2.90 (1.14)
Cortisol t2	3.98 (1.70)	3.68 (1.36)
Perceived stress scale t0	18.30 (8.46)	17.76 (6.95)
Perceived stress scale t1	16.05 (9.11)	13.62 (8.28)
Perceived stress scale t2	17.50 (7.94)	14.24 (7.83)
Attention and awareness t0	67.89 (10.24)	62.72 (12.02)
Attention and awareness t1	66.73 (9.45)	68.87 (7.26)
Attention and awareness t2	65.15 (12.41)	68.74 (12.18)
IANUS Job strain t0	49.25 (9.50)	51.47 (14.87)
IANUS Job strain t1	50.00 (11.58)	48.44 (18.32)
IANUS Job strain t2	50.50 (16.54)	45.88 (15.43)
IANUS Positive meaning t0	79.95 (14.91)	77.57 (14.83)
IANUS Positive meaning t1	82.19 (12.55)	77.73 (14.61)
IANUS Positive meaning t2	77.50 (14.96)	77.21 (14.14)
IANUS Emotional fatigue t0	40.83 (14.02)	49.51 (26.10)
IANUS Emotional fatigue t1	41.67 (11.79)	43.23 (26.57)
IANUS Emotional fatigue t2	35.00 (9.21)	42.16 (22.72)
IANUS Relational difficulties t0	43.33 (7.45)	47.55 (13.10)
IANUS Relational difficulties t1	42.92 (13.32)	46.35 (18.25)
IANUS Relational difficulties t2	44.58 (13.86)	44.12 (11.32)

Note: MBSR = Mindfulness-Based Stress Reduction; IANUS = Italian Cardiologists’ Undetected Distress Study.

**Table 3:** Model of the cortisol values in the control and MBSR groups at the 3 experimental times followed by post hoc tests.

	First part																	
	Cortisol—Within-Subjects Effects									df		F		*p*			η^2^_p_	
	Time									2		0.1		0.907			0	
	Time ✻ group									2		10.08		< 0.001			0.23	
	Residual									68								
	Between-Subjects Effects									df		F		*p*			η^2^_p_	
	Group									1		1.09		0.303			0.03	
	Residual									34								
	Cortisol Estimated Marginal Means—Time ✻ group										95% CI							
Group			Time						Mean	SE		Lower			Upper			
Control			T0						3.33	0.32		2.68			3.97			
			T1						4.61	0.35		3.9			5.33			
			T2						3.98	0.35		3.27			4.69			
Experimental			T0						4.09	0.36		3.37			4.82			
			T1						2.9	0.39		2.1			3.7			
			T2						3.68	0.39		2.89			4.47			
	Second part: Post hoc-tests																	
Time		Group 1			time		Group 2	Mean Difference				SE	t			*p*		p_holm_
T0		Control		–	T0		Exp.	−0.77				0.48	−1.6			0.118		0.886
T0		Control		–	T1		control	−1.29				0.34	−3.75			<.001		0.010 *
T0		Control		–	T1		Exp.	0.43				0.51	0.85			0.404		1
T0		Control		–	T2		control	−0.65				0.39	−1.7			0.098		0.886
T0		Control		–	T2		Exp.	−0.36				0.5	−0.7			0.486		1
T0		Exp.		–	T1		control	−0.52				0.5	−1.03			0.308		1
T0		Exp.		–	T1		Exp.	1.2				0.38	3.12			0.004		0.048 *
T0		Exp.		–	T2		control	0.11				0.5	0.23			0.823		1
T0		Exp.		–	T2		Exp.	0.41				0.43	0.96			0.345		1
T1		Control		–	T1		Exp.	1.72				0.53	3.24			0.003		0.037 *
T1		Control		–	T2		control	0.63				0.38	1.67			0.105		0.886
T1		Control		–	T2		Exp.	0.93				0.53	1.77			0.085		0.855
T1		Exp.		–	T2		control	−1.08				0.53	−2.06			0.047		0.567
T1		Exp.		–	T2		Exp.	−0.78				0.42	−1.85			0.073		0.806
T2		Control		–	T2		Exp.	0.3				0.52	0.57			0.571		1

Note. 34 degrees of freedom. Exp. = experimental MBSR group; * = *p* < 0.05.

**Table 4:** MAAS, attention and awareness and Post Hoc Comparisons.

Part 1															
Within-Subjects Effects						df			F			*p*	η^2^_p_		
Time						2			1.22			0.301	0.04		
Time ✻ group						2			4.51			0.015	0.12		
Residual						66									
Between-Subjects Effects						df			F			*p*	η^2^_p_		
Group						1			0.02			0.895	0.00		
Residual						33									
MAAS, Estimated Marginal Means—Time ✻ group									95% Confidence Interval						
group		Time		Mean				SE	Lower			Upper			
Control		T1		67.89				2.51	62.78			73.00			
		T2		66.73				1.94	62.79			70.67			
		T3		65.15				2.81	59.43			70.86			
experimental		T1		62.77				2.90	56.87			68.66			
		T2		68.73				2.24	64.17			73.28			
		T3		69.56				3.24	62.96			76.16			
Part 2: post hoc tests															
Time	group		Time		group		Mean Difference			SE	df		t	*p*	p_holm_
T0	control		T0		Exp.		5.12			3.83	33.00		1.34	0.191	1
			T1		control		1.16			1.99	33.00		0.58	0.564	1
			T1		Exp.		−0.84			3.36	33.00		−0.25	0.805	1
			T2		control		2.74			2.51	33.00		1.09	0.282	1
			T2		Exp.		−1.67			4.10	33.00		−0.41	0.687	1
	Exp.		T1		control		−3.96			3.49	33.00		−1.14	0.264	1
			T1		Exp.		−5.96			2.30	33.00		−2.60	0.014	0.210
			T2		control		−2.38			4.04	33.00		−0.59	0.559	1
			T2		Exp.		−6.79			2.89	33.00		−2.35	0.025	0.351
T1	control		T1		Exp.		−2.00			2.96	33.00		−0.67	0.505	1
			T2		control		1.58			1.94	33.00		0.81	0.421	1
			T2		Exp.		−2.83			3.78	33.00		−0.75	0.459	1
	Exp.		T2		control		3.58			3.59	33.00		1.00	0.326	1
			T2		Exp.		−0.83			2.24	33.00		−0.37	0.713	1
T2	control		T2		Exp.		−4.41			4.29	33.00		−1.03	0.311	1

**Table 5:** Results of repeated measures ANOVA on the Perceived Stress Scale.

Within-Subjects Effects	df		F	*p*	η^2^_p_		
Time	2		4.33	0.017	0.11		
time ✻ group	2		0.76	0.471	0.02		
Residual	68						
Between-Subjects Effects	df		F	*p*	η^2^_p_		
group	1		0.73	0.400	0.02		
Residual	34						
Estimated Marginal Means—Time ✻ group						95% CI	
Group	Time	Mean		SE		Lower	Upper
Control	T0	18.30		1.77		14.70	21.90
	T1	16.05		1.96		12.07	20.03
	T2	17.50		1.79		13.86	21.14
Experimental	T0	17.69		1.98		13.66	21.71
	T1	13.62		2.19		9.18	18.07
	T2	14.25		2.00		10.18	18.32

**Table 6:** Results of repeated measures ANOVA on the IANUS, Job strain.

Within-Subjects Effects	Df	F	*p*	η^2^_p_	
Time	2	0.71	0.493	0.02	
time ✻ group	2	1.74	0.183	0.05	
Residual	68				
Between-Subjects Effects	Df	F	*p*	η^2^_p_	
group	1	0.04	0.846	0.00	
Residual	34				
Estimated Marginal Means—Time ✻ group				95% CI	
Group	Time	Mean	SE	Lower	Upper
Control	T0	49.25	2.74	43.68	54.82
	T1	50.00	3.34	43.21	56.79
	T2	50.50	3.61	43.16	57.84
Experimental	T0	52.19	3.07	45.96	58.42
	T1	48.44	3.73	40.85	56.03
	T2	46.56	4.04	38.35	54.77

**Table 7:** Results of repeated measures ANOVA on the IANUS emotional fatigue with post hoc comparisons.

First part												
Within-Subjects Effects												
Effect			df		F	*p*			η^2^ₚ			
Time			2		6.71	0.002			0.16			
Time ✻ Group			2		2.96	0.059			0.08			
Residual			68									
Between-Subjects Effects												
Effect			df		F	*p*			η^2^ₚ			
Group			1		1.24	0.274			0.04			
Residual			34									
IANUS emotional fatigue, estimates marginal means—Time ✻ group												
Group			Time		mean	SE			95% CI Lower		95% CI Upper	
Control			T0		40.83	4.54			31.61		50.05	
			T1		41.67	4.41			32.70		50.63	
			T2		35.00	3.75			27.38		42.62	
Experimental			T0		51.04	5.07			40.73		61.35	
			T1		43.23	4.93			33.21		53.25	
			T2		43.23	4.19			34.71		51.75	
Second part: post hoc tests												
Time	Group	Time		Group	Mean Difference		SE	df		t	p	p_holm_
T0	Control	T0		Exp.	−10.21		6.81	34.0		−1.50	0.143	1
		T1		Control	−0.83		2.23	34.0		−0.37	0.711	1
		T1		Exp.	−2.40		6.70	34.0		−0.36	0.723	1
		T2		Control	5.83		2.70	34.0		2.16	0.038	0.413
		T2		Exp.	−2.40		6.18	34.0		−0.39	0.700	1
	Exp.	T1		Control	9.37		6.72	34.0		1.39	0.172	1
		T1		Exp.	7.81		2.49	34.0		3.13	0.004	0.053
		T2		Control	16.04		6.31	34.0		2.54	0.016	0.188
		T2		Exp.	7.81		3.01	34.0		2.59	0.014	0.181
T1	Control	T1		Exp.	−1.56		6.62	34.0		−0.24	0.815	1
		T2		Control	6.67		2.50	34.0		2.67	0.012	0.163
		T2		Exp.	−1.56		6.08	34.0		−0.26	0.799	1
	Exp.	T2		Control	8.23		6.19	34.0		1.33	0.193	1
		T2		Exp.	0.00		2.80	34.0		0.00	1.000	1
T2	Control	T2		Exp.	−8.23		5.62	34.0		−1.46	0.153	1

## Data Availability

The datasets presented in this article are not readily available because the data are part of a larger ongoing study. Requests to access the datasets should be directed to the corresponding author.

## Supplementary Materials

The following supporting information can be downloaded at: https://www.mdpi.com/article/10.3390/healthcare13192455/s1, power analysis.
