# Supplementary material for: Mindfulness Improves Awareness and Cortisol Levels During COVID-19 Lockdown: A Randomised Controlled Trial in Healthcare Workers
Source: Healthcare (Basel). 2025 Sep 27;13(19):2455. doi: 10.3390/healthcare13192455 (PMC12523626; doi:10.3390/healthcare13192455)
Supplement: Supplementary file 1 [file healthcare-13-02455-s001.zip › healthcare-3814465-supplementary.pdf]

Supplementary materials – power analysis

Because the sample size was fixed, a post hoc power analysis was performed for repeated measures ANOVA with a within-between interaction, assuming two groups, three repeated measurements, and a nonsphericity correction of 1. The parameters were: sample size = 37, correlation among repeated measures = 0.5, and significance level alpha = 0.01, adjusted for multiple comparisons (5 outcomes). The critical F value was 4.92, with numerator degrees of freedom = 2 and denominator degrees of freedom = 70.

For cortisol, the effect size f=0.55 (partial eta squared = 0.23) indicates a large effect. The achieved power was effectively 1.00, demonstrating very high sensitivity to detect effects of this size, given the sample and alpha level.

For the MAAS (measuring attention and awareness), the effect size f=0.37 (partial eta squared ≈ 0.12) corresponds to a medium effect, with an achieved power of 0.99, indicating excellent sensitivity to detect such effects.

For perceived stress, the effect size f=0.14 (partial eta squared ≈ 0.02) represents a very small effect. The achieved power was low at 0.22, indicating insufficient sensitivity to detect effects of this magnitude under the current conditions.

For job strain measured with the IANUS, the effect size f=0.23 (partial eta squared ≈ 0.05) indicates a small effect, with a modest power of 0.67, also reflecting insufficient sensitivity.

For emotional fatigue (IANUS), the effect size f=0.29 (partial eta squared ≈ 0.08) corresponds to a medium effect, and the power was high at 0.91, indicating good sensitivity to detect effects of this size.

These results support that post hoc power exceeds 0.80 for cortisol, attention and awareness, and emotional fatigue, confirming adequate power for these variables despite initial recruitment constraints. For the outcomes which were not statistically significant – perceived stress and job strain – the power did not reach the recommended threshold, but this does not seem problematic, as the results are not statistically significant.
